# Supplementary material for: Whole genome sequencing of Chinese clearhead icefish, Protosalanx hyalocranius
Source: Gigascience. 2017 Feb 24;6(4):1–6. doi: 10.1093/gigascience/giw012 (PMC5530312; doi:10.1093/gigascience/giw012)
Supplement: GIGA-D-16-00073_Original_Submission.pdf [file giw012_GIGA-D-16-00073_Original_Submission.pdf]

Manuscript
Click here to view linked References
Click here to download Manuscript manuscript-SCE_icefish.revised.docx

Whole genome sequencing of Chinese clearhead icefish, *Protosalanx hyalocranius*

Kai Liu[1]†, Dongpo Xu[1]†, Jia Li[2]†, Chao Bian[2]†, Jinrong Duan[1]†, Yanfeng Zhou[1]†, Minying Zhang[1], Xinxin You[2], Yang You[1], Jieming Chen[2], Hui Yu[2], Gangchun Xu[1], Di-an Fang[1], Jun Qiang[1], Shulun Jiang[1], Jie He[1], Junmin Xu[2,4,5], Qiong Shi[2,4,5,6*], Zhiyong Zhang[3*], Pao Xu[1,5*]

[1]Freshwater Fisheries Research Center, Chinese Academy of Fishery Sciences, Wuxi, 214081, China

[2]Shenzhen Key Lab of Marine Genomics, Guangdong Provincial Key Lab of Molecular Breeding in Marine Economic Animals, BGI, Shenzhen, 518083, China

[3]Institute of Oceanology &Marine Fisheries, Jiangsu, 226007, China

[4]BGI Zhenjiang Institute of Hydrobiology, Zhenjiang, 212000, China

[5]BGI Research Center for Aquatic Genomics, Chinese Academy of Fishery Sciences, Shenzhen, 518083, China

[6]Laboratory of Aquatic Genomics, College of Ecology and Evolution, School of Life Sciences, Sun Yat-Sen University, Guangzhou, 510275, China

† Equal contributors

* Correspondence: xup@ffrc.cn (PX); shiqiong@genomics.cn (QS); 13906292412@139.com (ZZ)

Email addresses: liuk@ffrc.cn (KL); xudp@ffrc.cn(DX); lijia1@genomics.cn (JL); bianchao@genomics.cn (CB); duanjr@ffrc.cn(JD); zhouyf@ffrc.cn(YZ); zhangmy@ffrc.cn(MZ); youxinxin@genomics.cn (XY); youy@ffrc.cn (YY); chenjieming@genomics.cn (JC); yuhui@genomics.cn (HY); xugc@ffrc.cn(GX); fangda@ffrc.cn(DF); qiangj@ffrc.cn(JQ); 420219380@qq.com(SJ); hej@ffrc.cn(JH); xujunmin@genomics.cn (JX); shiqiong@genomics.cn (QS); 13906292412@139.com (ZZ); xup@ffrc.cn (PX)

Abstract

Background: Chinese clearhead icefish, *Protosalanx hyalocranius,* is a representative species of icefishes with economic importance and special appearance. Due to its great economic value in China, the fish was introduced to Lake Taihu and several other lakes half a century ago. Similar to the *Sinocyclocheilus* cavefishes, the clearhead icefish also has certain cavefish-like traits, such as transparent body and nearly scaleless skin. Here, we provided the whole genome sequence of this surface-dwelling fish and generated a high-quality genome assembly, aiming at exploring molecular mechanisms for these biological characteristics.

Findings: A total of 252.1 gigabases (Gb) of raw reads were sequenced. Subsequently, a novel high-quality genome assembly was generated, with the scaffold N50 reaching 1.163 Mb. The genome completeness was estimated to be 98.39% by using CEGMA and BUSCO evaluation. Finally, we annotated 19,884 protein-coding genes and observed that repeat sequences account for 24.43% of the genome assembly.

Conclusion: We report the whole genome sequencing of the Chinese clearhead icefish. The assembled genome will provide a significant foundation for further

molecular breeding and germplasm resource protection in the clearhead icefish, as well as other icefishes. It is also a valuable genetic resource for revealing the molecular mechanisms for the cavefish-like characteristics.

Keywords: Icefish; *Protosalanx hyalocranius*; Whole genome sequencing; Genome assembly; Gene prediction; Repetitive sequences

Data description

Background

Icefishes (Osmeriformes, Salangidae) are widely distributed in freshwater, coastal and estuarine habitats in East Asian countries [1-3]. Chinese clearhead icefish (*Protosalanx hyalocranius*), a diadromous fish, mainly inhabits in coastal areas and adjacent freshwaters [4-6]. As a commercially important fish in China, the clearhead icefish was widely introduced into some lakes half a century ago and has developed a resident life history [2, 7, 8]. Because of its transparent body and nearly scaleless skin, similar to the *Sinocyclocheilus* cavefishes [9], we are very interested in this surface-dwelling fish and are performing comparative genomics studies to explore the mechanisms for these biological phenotypes. However, with the rapid development of the Chinese economy in recent decades, population size of the clearhead icefish has been seriously declining because of overfishing, construction of water conservancy facilities and water pollution in the ecological systems [10]. To maintain its sustainable development in China, here we performed the genome sequencing of Chinese clearhead icefish for its biological and economic importance.

Sample and Sequencing

In this study, we applied Illumina whole genome sequencing strategy to generate the genome of Chinese clearhead icefish (NCBI Taxonomy ID: 418454; Fishbase ID: 12236). Genomic DNAs were isolated from the muscle tissue of an individual

collected from the Lake Taihu of Jiangsu Province in China. We constructed seven paired-end libraries with three short-insert libraries (250, 500 and 800 bp) and four long-insert libraries (2, 5, 10 and 20 kb) using the standard protocol provided by Illumina (San Diego, USA). Subsequent paired-end sequencing was performed by the Illumina HiSeq 2000 platform for each library. Finally, we obtained 252.1 Gb of raw 125-bp reads for further analysis.

Genome size estimation and genome assembly

The SOAPfilter v2.2 software [11] with optimized parameters (-y -p -g 1 -o clean -M 2 -f 0) was performed to remove low-quality row reads (including reads with 10 or more Ns and low-quality bases) and PCR-replicates as well as adaptor sequences. In total, we obtained 169.0 Gb of clean reads. Subsequently, we estimated the genome size based on the 17-mers depth frequency distribution method [12]. A 17-mer represents an artificial division with 17-bp length nucleotide segment of sequencing reads, therefore, a raw sequence read with a total length of L bp contains (L-17+1) 17-mers. The genome size was estimated with the following formula: $G = N*(L-17+1)/K\text{-depth}$, in which G is the genome size, N is the total number of reads, and K-depth is the highest frequency of 17-mer analysis. In our current study, N was 10,500,000,000 and the K-depth was 20. Hence, we estimated that the genome size of Chinese clearhead icefish is 525 Mb.

The filtered reads were assembled using SOAPdenovo2 v2.04.4 software [13] with optimized parameters (pregraph -K 79 -d 1; contig -M 1; scaff -F -b 1.5 -p 16) to generate contigs and original scaffolds. The gaps were fulfilled using GapCloser v1.12 software [14] with default parameters and –p set to 25. Finally, we generated a high-quality genome assembly of 536 Mb, with the scaffold N50 reaching 1.163 Mb (Table 1).

The completeness of our assembly was evaluated by using CEGMA [15] and BUSCO [16]. The CEGMA program (Core Eukaryotic Genes Mapping Approach; version 2.4)

assessment with 248 conserved Core Eukaryotic Genes (CEGs) was performed for evaluation of the gene space completeness. The results revealed that the assembled genome had a CEGMA completeness score about 90.32% and 98.39%, which was calculated from the complete gene set and the partial gene set respectively. Meanwhile, we used the representative metazoa gene set [17], which contains 843 single-copy genes that are widely present in metazoa. The assessment demonstrated that the BUSCO values is 89%, containing [D: 10%], F: 7.7%, M: 2.9%, n: 843 (C: complete [D: duplicated], F: fragmented, M: missed, n: genes). These data from CEGMA and BUSCO indicate that the assembled genome covered majority of the gene space.

Repeat annotation

Firstly, a *de novo* repeat library was constructed by the RepeatModeller v1.05 [18] and LTR_FINDER.x86_64-1.0.6 [10] with default parameters. Then, our assembly genome sequences were aligned against the ReBase v21.01 [19] and the *de novo* repeat libraries to recognize the known and novel TEs (transposable elements) using the RepeatMasker v4.06 [20]. Meantime, the Tandem Repeat Finder v4.07 [21] with parameters “Match=2, Mismatch=7, Delta=7, PM=80, PI=10, Minscore=50, and MaxPerid=2000” was utilized to annotate tandem repeats. Furthermore, the RepeatProteinMask software v4.0.6 [20] was used to predict TE relevant proteins in our genome assembly. Finally, we observed that the repeat sequences account for 24.43% of the assembled genome (Table 1).

Genome Annotation

In brief, we utilized two different methods to predict total gene set of the clearhead icefish.

1) *de novo* annotation. The AUGUSTUS v2.5 [22] and GENSCAN v1.0 [23] were executed to *ab initio* predict genes within the assembled genome, with the repetitive

sequences masked as “N” in order to discard pseudo gene prediction. Those low-quality genes with short length (<150 bp), premature termination or frame-shifting were removed.

2) Homology annotation. We aligned the protein sequences from six published genomes, including *Danio rerio* [24], *Oryzias latipes* [25], *Takifugu rubripes* [26], *Tetraodon nigroviridis* [27], *Esox lucius* [28] and *Gasterosteus aculeatus* [29], against our assembly to predict homology-based genes. The potential homology-based genes were searched by TblastN [30] with an e-value of 10^{-5}. The TblastN results were then processed by SOLAR (Sorting Out Local Alignment Result [31]) to obtain the best hit of each alignment. Subsequently, GeneWise v2.2.0 [32] was performed to detect the possible gene structure for the best hit of each alignment. The low-quality genes were also removed as described in the above-mentioned *do novo* annotation.

3) Integration of annotation results. To merge all results produced from the above methods, we employed the GLEAN [33] to generate a non-redundant and comprehensive gene set. Finally, the best hit of each protein was obtained through all protein sequences from the GLEAN results aligned to the databases of the SwissProt and TrEMBL [34] (Uniprot release 2011.06) by BlastP with an e-value of 10^{-5}. Overall, we generated a final gene set with 19,884 genes for the Chinese clearhead icefish.

CEGMA was performed again to evaluate the coverage rate between KOG (EuKaryotic Orthologous Groups) genes predicted by CEGMA and the predicted total gene set. It demonstrates that the predicted gene set mapped 96.4% of the KOGs. Simultaneously, the BUSCO was implemented again to assess the completeness of the predicted gene set. The BUSCO values were calculated as follows: C: 79% [D: 16%], F: 9.8%, M: 10%, n: 843 (C: complete [D: duplicated], F: fragmented, M: missed, n: genes). The assessment values from both CEGMA and BUSCO proved high accuracy of the annotation.

4) Function annotation. The predicted protein sequences of clearhead icefish were aligned against several public databases (Pfam [35], PRINTS [36], ProDom [37] and SMART [38]) for detection of functional motifs and domains . Finally, we found that 96.2% of the predicted total gene set had been annotated with at least one functional assignment from other public databases (Swiss-Prot [39], Interpro [40], TrEMBL [41] and KEGG [42]).

Conclusion

We generated a high-quality genome assembly of Chinese clearhead icefish. The novel genome data were deposited in publicly accessible repositories to promote further biological research, molecular breeding and resource protection of this representative icefish.

Availability of supporting data

Supporting data are available in the GigaDB database [cite when ready], and the raw whole genome sequences are deposited in the SRA under bioproject number PRJNA328051.

Competing interests

The authors declare that they have no competing interests.

Funding

This study was supported by a grant from fish investigation in Taihu Lake (No. TH2016WT007), National Infrastructure of Fishery Germplasm Resources (No. 2016DKA30470), Basic Research Funds from Freshwater Fisheries Research Center (No. 2013JBFM07), Special Project on the Integration of Industry, Education and Research of Guangdong Province (No. 2013B090800017), Shenzhen Special Program

for Future Industrial Development (No. JSGG20141020113728803), and Zhenjiang Leading Talent Program for Innovation and Entrepreneurship.

Author's Contributions

KL, PX, QS, DX, JX, CB and ZZ conceived the project. MZ, XY, HY, JC, GX, DF, JQ, SJ and JH collected the samples and extracted the genomic DNA. JL, CB and HY performed the genome assembly and data analysis. KL, XP, JL, CB, QS, KL, YY and ZZ wrote the paper.

References

1. Wang ZS, Cui Zhang FU: **Biodiversity of Chinese Icefishes (Salangidae) and their conserving strategies**. *Chinese Biodiversity* 2002, **10**(4):416-424.
2. Zhang J, Li M, Xu M, Takita T, Wei F: **Molecular phylogeny of icefish Salangidae based on complete mtDNA cytochrome b sequences, with comments on estuarine fish evolution**. *Biological Journal of the Linnean Society* 2007, **91**(2):325-340.
3. Wang Z, Lu C, Hu H, Xu C, Lei G: **Dynamics of Icefish (Salangidae) Stocks in Nanyi Lake, Eastern China: Degradation and Overfishing**. *Journal of Freshwater Ecology* 2004, **19**(2):271-278.
4. Xia DQ, Cao Y, Ting ting WU, Yang H: **Study on lineages of Protosalanx chinensis, Neosalanx taihuensis and N.oligodontis in Taihu Lake with RAPD technique**. *Journal of Fisheryences of China* 2000, **7**(01):12-15.
5. Xia DQ, Cao Y, Ting Ting WU, Yang H: **Genetic Structures of Population of Protosalanx Chinensis, Neosalanx Taihuensis and Neosalanx Oligodontis in Lake Taihu**. *Journal of Fisheries of China* 1999(03):254-260.
6. Armani A, Castigliego L, Tinacci L, Gianfaldoni D, Guidi A: **Molecular characterization of icefish, (S alangidae family), using direct sequencing of mitochondrial cytochrome b gene**. *Food Control* 2011, **22**(6):888-895.
7. Wang Z, Lu C, Hu H, Zhou Y, Xu C, Lei G: **Freshwater icefishes (Salangidae) in the Yangtze River basin of China: Spatial distribution patterns and environmental determinants**. *Environmental Biology of Fishes* 2005, **73**(3):253-262.
8. Ye S, Yang J, Liu H, Oshima Y: **Use of elemental fingerprint analysis to identify localities of collection for the large icefish protosalanx chinensis**

in Taihu Lake, China. *Journal of the Faculty of Agriculture, Kyushu University* 2011, **56**(1):41-45.

9. Yang J, Chen X, Jie B, Fang D, Ying Q, Jiang W, Hui Y, Chao B, Jiang L, He S: **The Sinocyclocheilus cavefish genome provides insights into cave adaptation**. *Bmc Biology* 2016, **14**(1):1-13.
10. Xu J, Xie P, Zhang M, Zhou Q, Zhang L, Wen Z, Cao T: **Icefish (salangidae) as an indicator of anthropogenic pollution in freshwater systems using nitrogen isotope analysis**. *Bulletin of environmental contamination and toxicology* 2007, **79**(3):323-326.
11. **SOAPfilter v2.2 software:** http://soap.genomics.org.cn/index.html.
12. Liu B, Shi Y, Yuan J, Hu X, Zhang H, Li N, Li Z, Chen Y, Mu D, Fan W: **Estimation of genomic characteristics by analyzing k-mer frequency in de novo genome projects**. *Quantitative Biology* 2013, **35**(s 1–3):62-67.
13. Luo R, Liu B, Xie Y, Li Z, Huang W, Yuan J, He G, Chen Y, Pan Q, Liu Y *et al*: **SOAPdenovo2: an empirically improved memory-efficient short-read de novo assembler**. *Gigascience* 2012, **1**:18.
14. Li R, Yu C, Li Y, Lam TW, Yiu SM, Kristiansen K, Wang J: **SOAP2: an improved ultrafast tool for short read alignment**. *Bioinformatics* 2009, **25**(15):1966-1967.
15. Parra G, Bradnam K, Korf I: **CEGMA: a pipeline to accurately annotate core genes in eukaryotic genomes**. *Bioinformatics* 2007, **23**(9):1061-1067.
16. Sim AFO, Waterhouse MR, Ioannidis P, Kriventseva VE, Zdobnov ME: **BUSCO: assessing genome assembly and annotation completeness with single-copy orthologs**. *Bioinformatics* 2015, **31**(19):3210-3212.
17. Kriventseva EV, Tegenfeldt F, Petty TJ, Waterhouse RM, Simão FA, Pozdnyakov IA, Ioannidis P, Zdobnov EM: **OrthoDB v8: update of the hierarchical catalog of orthologs and the underlying free software**. *Nucleic Acids Research* 2015, **43**(Database issue):D250-D256.
18. **RepeatModeller v1.05 software:** http://www.repeatmasker.org/RepeatModeler.html.
19. J J, VV K, A P, P K, O K, J W: **Repbase Update, a database of eukaryotic repetitive elements**. *Cytogenetic & Genome Research* 2005, **110**(1-4):462-467.
20. Chen N: **Using RepeatMasker to Identify Repetitive Elements in Genomic Sequences**: John Wiley & Sons, Inc.; 2004.
21. Benson G, . **Tandem repeats finder: a program to analyze DNA sequences**. *Nucleic Acids Research* 1999, **27**(2):573-580(578).
22. Mario S, Oliver K, Irfan G, Alec H, Stephan W, Burkhard M: **AUGUSTUS: ab initio prediction of alternative transcripts**. *Nucleic Acids Research* 2006, **34**(WebServerissue):435-439.
23. Burge C, ., Karlin S, . **Prediction of complete gene structures in human genomic DNA**. *Journal of Molecular Biology* 1997, **268**(1):78-94.

24. Collins JE, White S, Searle SMJ, Stemple DL: **Incorporating RNA-seq data into the zebrafish Ensembl genebuild**. *Genome Research* 2012, **22**(10):2067-2078.
25. Kasahara M, Naruse K, Sasaki S, Nakatani Y, Qu W, Ahsan B, Yamada T, Nagayasu Y, Doi K, Kasai Y: **The medaka draft genome and insights into vertebrate genome evolution**. *Nature* 2007, **447**(7145):714-719.
26. Kesteven GL: **Whole-Genome Shotgun Assembly and Analysis of the Genome of Fugu rubripes**. *Science (New York, NY)* 2002, **297**(5585):1301-1310.
27. Jaillon O, Aury JM, Brunet F, Petit JL, Stangethomann N, Mauceli E, Bouneau L, Fischer C, Ozoufcostaz C, Bernot A: **Genome duplication in the teleost fish Tetraodon nigroviridis reveals the early vertebrate proto-karyotype**. *Nature* 2004, **431**(7011):946-957.
28. Rondeau EB, Minkley DR, Leong JS, Messmer AM, Jantzen JR, von Schalburg KR, Lemon C, Bird NH, Koop BF: **The genome and linkage map of the northern pike (Esox lucius): conserved synteny revealed between the salmonid sister group and the Neoteleostei**. *PLoS ONE* 2014, **9**(7):e102089.
29. Jones FC, Grabherr MG, Chan YF, Russell P, Mauceli E, Johnson J, Swofford R, Pirun M, Zody MC, White S: **The genomic basis of adaptive evolution in threespine sticklebacks**. *Nature* 2012, **484**(7392):55-61.
30. Pevsner J: **Basic Local Alignment Search Tool (BLAST)**: John Wiley & Sons, Inc.; 2005.
31. Yu XJ, Zheng HK, Wang J, Wang W, Su B: **Detecting lineage-specific adaptive evolution of brain-expressed genes in human using rhesus macaque as outgroup**. *Genomics* 2006, **88**(6):745-751.
32. Birney E, Clamp M, Durbin R: **GeneWise and Genomewise**. *Genome Research* 2004, **14**(5):988-995.
33. Elsik CG, Mackey AJ, Reese JT, Milshina NV, Roos DS, Weinstock GM: **Creating a honey bee consensus gene set**. *Genome Biology* 2007, **8**(1):90-105.
34. Bairoch A, Apweiler R: **The SWISS-PROT protein sequence database and its supplement TrEMBL in 2000**. *Nucleic Acids Res* 2000, **28**(1):45-48.
35. Finn RD: **Pfam: the protein families database**. *Nucleic Acids Research* 2014, **42**(Database issue):D222-230.
36. Attwood TK: **The PRINTS database: A resource for identification of protein families**. *Briefings in Bioinformatics* 2002, **3**(3):252-263.
37. Bru C, Courcelle E, Beausse Y, Dalmar S, Kahn D: **The ProDom database of protein domain families: more emphasis on 3D**. *Nucleic Acids Research* 2005, **33**(Database issue):212-215.

38. Letunic I, Copley RR, Schmidt S, Ciccarelli FD, Doerks T, Schultz J, Ponting CP, Bork P: **SMART 4.0: towards genomic data integration**. *Nucleic Acids Research* 2004, **32**(Databaseissue):D142-D144.
39. B B, A B, R A, MC B, A E, E G, MJ M, K M, C OD, I P *et al*: **The Swiss-Prot knowledgebase and its supplement TREMBL in 2003**. *Nucleic Acids Research* 2003, **31**(1):365-370.
40. Hunter S, Apweiler R, Attwood TK, Bairoch A, Bateman A, Binns D, Bork P, Das U, Daugherty L, Duquenne L: **InterPro: the integrative protein signature database**. *Nucleic Acids Research* 2009, **37**(suppl 1):D211-D215.
41. Hingamp P, Broek AEVD, Stoesser G, Baker W: **The EMBL nucleotide sequence database**. *Molecular Biotechnology* 1999, **12**(3):255-267.
42. Kanehisa M, Goto S: **KEGG: kyoto encyclopedia of genes and genomes**. *Nucleic Acids Research* 2000, **27**(1):29-34(26).

Tables

Table 1. The statistics of genome assembly and annotation for *P. hyalocranius*.

Genome assembly	
Contig N50 size (kb)	17.2
Scaffold N50 size (Mb)	1.163
Estimated genome size (Mb)	525
Assembled genome size (Mb)	536
Genome coverage (X)	315
The longest scaffold (bp)	5,398,389
Genome annotation	
Protein-coding gene number	19,884
Annotated functional gene number	19,125 (96.2%)
Unannotated functional gene number	759 (3.8%)
Repeat content	24.43%
