# Supplementary material for: Whole genome sequencing of Chinese clearhead icefish, Protosalanx hyalocranius
Source: Gigascience. 2017 Feb 24;6(4):1–6. doi: 10.1093/gigascience/giw012 (PMC5530312; doi:10.1093/gigascience/giw012)
Supplement: Response_to_Reviewer_Comments_Original_Submission.pdf [file giw012_Response_to_Reviewer_Comments_Original_Submission.pdf]

Oct 12, 2016
RE: Your submission to GigaScience - GIGA-D-16-00073

Dear Hans,
Thanks for your advice and the comments of reviewers.

We made revisions based on your suggestions. Related point-by-point responses (in the blue color) to the comments of reviewers are provided as follows for your consideration.

By the way, the queries related to the discarded sequences and a potential error in our genome size calculation, from the reviewer 1, were answered in detail. We also added a new table (Table 2; to summarize the classification of repeat sequences), a phylogenetic tree (Figure 1) and the whole genome duplication analyses (lines 166-206 in the main text and Figure 2), in accordance with the opinions from the reviewer 2.

Best regards,
Qiong Shi, PhD, Professor
BGI
Shenzhen 518083
China

P.S. Our point-by-point responses (in the blue color) to the comments of reviewers:

Reviewer #1: This manuscript describes the genome assembly of the intriguing Chinese clearhead icefish. Overall, the sequencing and assembly meet the standards for a genome based on Illumina technology, as do the annotation and validation.
There are a few issues I would like to ask the authors to clarify:
1) Is it correct that a full third of the original sequencing data was discarded (252.1 Gbp -> 169.0 Gbp)? I could not find the exact meaning of SOAPfilter settings. (I think this tool does not include k-mer-based error correction or read trimming?)

Answer: Yes, we are really convinced of the filtering steps and the data of sequenced reads. In fact, we took necessary filtering processes to remove 3 and 1 low-quality bases in left and right edges of the raw reads and to discard those duplicated reads produced by sequencing PCRs. Usually, around 20-30% of raw reads were removed in our previous reports (You et al. 2014, Nature Communications, 5:5594; Yang et al., 2015, BMC Biology, 14:1; Bian et al., 2016, Scientific Reports, 6:24501; Chen et al., 2016, GigaScience, 5:39; Lin et al., 2016, Nature, in press) when the same processes were applied. For our present work, the discarding percentage is a little bit higher because we generated much more sequence (over 300×; without beforehand estimation of the genome size) than the necessary 150~200×, therefore, the parameters are more stringent so as to obtain a much better assembly. We indeed estimated the genome size (~0.5 Gb) with the k-mer analysis (lines 85-96 and Table 1).

SOAPfilter contains the trimming process that can remove those reads with both edges of low-quality bases. The input file for the SOAPfilter likes: 150822_I178_FCC7F9KANXX_L2_WHPROfreDAABDLAAPEI-106_1.fq.gz 3 1 40 or
150822_I178_FCC7F9KANXX_L2_WHPROfreDAABDLAAPEI-106_2.fq.gz 3 1 10.
150822_I178_FCC7F9KANXX_L2_WHPROfreDAABDLAAPEI-106_1.fq.gz stands for the file of reads, the followed 3 and 1 represent that the SOAPfilter will discard reads with 3 and 1 low-quality bases in the left and right edges, respectively. The last number, 40 or 10, indicates that the SOAPfilter software will discard the reads with over 40% low-quality bases or with more than 10 Ns (not determined sequence bases).

2) The reason I ask, is because the genome size calculations (lines 97-101) are incorrect. Given N = 10.5 billion, k-depth = 20, it is easy to see how the 525 Mbp genome size was derived. However, the formula is

not G = N/k-depth, and there should have been only 2 billion original reads, so this is clearly not the read number. Calculating N using the correct formula (line 98), I get 525 million = N * (125-17+1)/20, so N = 96 million, which is also nowhere near the (filtered) number of reads. Was a subset used? (Also note that the formula is only valid if all reads are of identical length, therefore trimmed reads should be omitted). In any case, a k-mer depth of only 20 must be incorrect (or based on a subset) in itself, as the genome coverage (table 1) is 315x.

Answer: Thank you for the question, which may be generated by our ambiguous statements regarding the N values for calculation of the estimated genome size. Hence, we rewrote the related section in the revised manuscript (lines 92-96) to make clear statements.
In fact, as we know, the start positions of sequenced reads follow a Poisson distribution pattern. When the read length (L) is far shorter than the genome size (L<
Based on the Poisson theory, we actually applied the following formula to calculate the genome size: G=K_num/K_depth=b_num/b_depth. K_num is the total number of K-mers from the sequencing data, K_depth is the expected coverage depth for k-mers, b_num is the total number of bases, b_depth is the expected coverage depth of bases; As one read with length L generates L-K+1 k-mers, K_num /b_num = (L-K+1) / L. In our manuscript (lines 92-96), the K_num was 10, 500,000,000, and the K_depth was 20. Therefore, the estimated genome size is 525 Mb.
Reference: Liu B, Shi Y, Yuan J, Hu X, Zhang H, Li N, Li Z, Chen Y, Mu D, Fan W: Estimation of genomic characteristics by analyzing k-mer frequency in de novo genome projects. Quantitative Biology 2013, 35(s 1–3):62-67.

3) Line 106: it should be reported that the 536 Mbp in scaffolds contain 121.7 Mbp in gaps. Whether the assembly then still qualifies as high quality is debatable, this depends fully on whether the genome size is really expected to be 525 Mbp (in which case the assembly misses 21% of the genome - not high quality), or whether the genome size is actually much smaller and the gaps between contigs are artificially large because of uncertainties in read library insert sizes.

Answer: Thanks for your comment. Our assembly has a long contig at 17.2 kb, which was sufficient for the further genome analyses, including annotation and evolution discussion. However, “high quality” should be rewritten; hence, we change it to be “a draft genome”.
It is the first version for the clearhead icefish genome assembly, and we will sequence more with Pacbio to improve the assembly quality of this valuable fish.

Typos:
Line 104: 'fulfilled' -> better 'filled'
Line 124: ReBase -> RepBase. Also, please fix the author list of the corresponding entry [19] in the references.
Line 142: 'six … genomes, including…' then lists all six.

Answer: Thanks for your nice suggestions. We revised these sentences according to your advice.

Reviewer #2: The paper presented here provides efficient combination of different programs used to characterise the genome of the Chinese clear head icefish.
I only can regret that the basic results provided here are way too succinct to get a full appreciation for the reviewers and the readers, lately. As an example, we are left with an final average value for the total number of transposable elements in this genome, but we are left with no idea about the proportions of each TE subdivisions. No evolutionary values are provided. Teleost fish are known to have a extra round of whole genome duplication, this result was not searched, nor discussed. Synteny hasn't been considered as well.
The authors put forward the methods they use and they provide minimalist details of the results.
Why hasn't the homology annotation done using the tilapia and platyfish?
Having in hand such a genome could have been the opportunity for more results, to enhance the interest of this publication. As an example, some phylogenetical analyses of key genes.

Answer: Thanks for your instructive suggestions. We really agree with you that providing the detailed classification of repeat sequences and adding the sections of synteny blocks and phylogentical tree will enhance the interests of readers. Therefore, we provide detailed descriptions of these three areas (lines 115-126 & 166-206) and related Table 2 and Figures 1 & 2 in the revised manuscript.
On the other hand, we selected six representative species, including Danio rerio, Oryzias latipes, Takifugu rubripes, Tetraodon nigroviridis, Esox lucius and Gasterosteus aculeatus, to perform the homolog annotation. As reported in our previous genome papers (mudskipper: You et al., 2014; channel catfish: Chen et al., 2016), genome data from these six species were sufficient for gene annotation. Therefore, the final predicted gene set in our present icefish work, evaluated by the BUSCO software, was indeed relatively complete.
Reference:
1.You X, Bian C, Zan Q, Xu X, Liu X, Chen J, Wang J, Qiu Y, Li W, Zhang X et al: Mudskipper genomes provide insights into the terrestrial adaptation of amphibious fishes. Nature communications 2014, 5:5594.
2.Chen X, Zhong L, Bian C, Xu P, Qiu Y, You X, Zhang S, Huang Y, Li J, Wang M et al: High-quality genome assembly of channel catfish, Ictalurus punctatus. GigaScience 2016, 5(1):39.

Minor typos:
l15 Missing blank space
l42 no plural at cavefish (fishes only used when an exact number is provided)
l124 missing "p" in RepBase
l128 missing "o" in MaxPeriod
l151 we emplyed GLEAN (no need of article before GLEAN)
The bibliography has a strong record of missing names (e.g. ref 19 and 39, or layout problems, like in ref. 20, 21, 23)

Answer: Thanks for your advice. We have corrected these issues according to your instructions.
