# Supplementary material for: Whole genome sequencing of Chinese clearhead icefish, Protosalanx hyalocranius
Source: Gigascience. 2017 Feb 24;6(4):1–6. doi: 10.1093/gigascience/giw012 (PMC5530312; doi:10.1093/gigascience/giw012)
Supplement: Response_to_Reviewer_Comments_Revision_1.pdf [file giw012_Response_to_Reviewer_Comments_Revision_1.pdf]

Nov 2, 2016
RE: Your submission to GigaScience - GIGA-D-16-00073

Dear Hans,
Thanks for your advice and the instructive comments from both reviewers.

We made revisions based on your suggestions. Related point-by-point responses to the comments of reviewers are provided as follows for your consideration.

By the way, we used Nile tilapia as the reference to perform the 4DTV analysis again for identification of the WGD incident (Figure 2) and revised the corresponding sentences (Section: Synteny blocks and genome duplication) in the revised manuscript (lines 189-206). We also changed the species names to the names of taxonomic groups within the revised phylogenetic tree (Figure 1).

Best regards,
Qiong Shi, PhD, Professor
BGI
Shenzhen 518083
China

P.S. Our point-by-point responses to the comments of reviewers:

Reviewer reports:
Reviewer #1: I thank the authors for responding to the minor issues I raised, and for their inclusion of additional evolutionary analyses. I am satisfied with their responses, however I still have a few minor suggestions:
- Regarding the genome size calculation, thank you for the clarification. As I read the formula, it is now clear that this calculation is based on a subset of the data. This is of course fine (and wise), but to avoid confusion I would suggest explicitly stating this in line 88 or 95.

Answer: Thanks for your nice suggestion. We corrected these sentences in our revised manuscript (lines 89-94).

- Regarding the assembly size, I think the total gap length could be included in table 1.

Answer: Yes, it is included in the revised Table 1.

As for the evolutionary analyses, these are interesting and appropriate, however in both cases I suggest rephrasing the final lines summarizing the results, as these are now slightly ambiguous.
- Line 185/186: 'the close relationship between clearhead icefish and zebrafish & medaka'. Zebrafish and medaka are themselves not closely related fish species at all. Perhaps it is better to simply state that the data demonstrate the phylogenetic position of the clearhead icefish.

Answer: Thanks for your advice. We modified the description (lines 185-186) of phylogenetic tree according to your suggestion.

-Line 206: 'clearhead icefish also experienced the WGD, and it appeared more recently than medaka'. This could be read as a more recent WGD than in the case of medaka, when it is of course the same WGD (at exactly the same time).

Answer: Thanks for your comment. We changed the related sentence of WGD incident in lines 204-206. We also used Nile tilapia as the reference, instead of the previous medaka for calculation of 4DTV distances, because Nile tilapia owns a more completed gene set.

Reviewer #2: I have not much to say about the quality of the work mastered here by the authors to generate the assembly of another fish genome, but among the Stomatii, a taxonomic location not yet been investigated.
Sentence line 185-186 is not well written. The phylogeny does not match the statement. Maybe the authors should rather name the taxonomic groups rather than naming two species that twisted their meaning.

Answer: Thanks for your suggestion. We changed the species names to their corresponding names of taxonomic groups in the phylogenetic tree (Figure 1).

Sentence line 204-206. This sentence shows that the authors have been mislead. There was only one event of whole genome duplication at the base of the Teleost genome. The different average rate of evolution in the different species observed by gene comparison has a different meaning that the one stated here. In this case, it is more suitable to rephrase this sentence. See similar comparisons made in the trout genome paper by Berthelot et al. 2014. Therefore, fig.2 is just a glimpse of what can be done, and this interpretation is too scarce and misleading.
Besides, recently published genomes have a finer tuned gene descriptions as better annotated sequences and more genes are described. Oreochromis niloticus, the Nile tilapia is a good example. On the contrary, the medaka genome lacks several genes, found in several other fish species, and sometimes key genes.
One suggestion would be to find out the number of duplicates originating from the teleost fish duplications compared to what have been described in the other species.

Answer: Thanks for your instructive suggestion. We used Nile tilapia as the reference to perform the analysis of WGD again. We also revised the related sentence to describe the WGD incident of clearhead icefish (lines 204-206). On the other hand, the distribution of 4DTV is indeed professional method for identification of the WGD incidents. It has been wildly used in massive genome papers, such as common carp [1], Japanese lampery [2], etc. Our new result of 4DTV shows that the clearhead icefish experienced the same WGD incident as Nile tilapia.
[1] Xu P, Zhang X, Wang X, Li J, Liu G, Kuang Y, Xu J, Zheng X, Ren L, Wang G et al: Genome sequence and genetic diversity of the common carp, Cyprinus carpio. Nature genetics 2014, 46(11):1212-1219.
[2] Mehta T K, Ravi V, Yamasaki S, et al. Evidence for at least six Hox clusters in the Japanese lamprey (Lethenteron japonicum). Proceedings of the National Academy of Sciences, 2013, 110(40): 16044-16049.

What TRF stands for in Table 2?

Answer: TRF represents the Tandem Repeat Finder. We provided its full name in the revised Table 2.

Line 184 typo in the word "sequence".
several "&" instead of "and".

Answer: Thanks for your advice. We have corrected these errors according to your instructions.
