# Supplementary material for: Whole genome sequencing of Chinese clearhead icefish, Protosalanx hyalocranius
Source: Gigascience. 2017 Feb 24;6(4):1–6. doi: 10.1093/gigascience/giw012 (PMC5530312; doi:10.1093/gigascience/giw012)
Supplement: Resposne_to_Reviewers_Comments_Revision_3.pdf [file giw012_Resposne_to_Reviewers_Comments_Revision_3.pdf]

Dear editor Hans,

Thanks for your letter.

In accordance with your request, we added a picture of Chinese clearhead icefish as Figure 1 of our manuscript.

Please do not hesitate to contact us, if you have any query.
Best regard
