# Supplementary material for: Whole genome sequencing of Chinese clearhead icefish, Protosalanx hyalocranius
Source: Gigascience. 2017 Feb 24;6(4):1–6. doi: 10.1093/gigascience/giw012 (PMC5530312; doi:10.1093/gigascience/giw012)
Supplement: Resposne_to_Reviewer_Comment_Revision_2.pdf [file giw012_Resposne_to_Reviewer_Comment_Revision_2.pdf]

Nov.17th, 2016
RE: Your submission to GigaScience - GIGA-D-16-00073

Dear editor Hans,

Thanks for your letter. We made revisions based on your suggestions.

We added the detailed table (Table 3: Gene annotation statistics of the genome of P. hyalocranius.) to demonstrate the annotation result in each step containing the denovo annotation (AUGUSTUS and GENSCAN), homolog annotation and Glean process.

Best regards,
Qiong Shi, PhD, Professor
BGI
Shenzhen 518083
China
