# Supplementary material for: Whole genome sequencing of Chinese clearhead icefish, Protosalanx hyalocranius
Source: Gigascience. 2017 Feb 24;6(4):1–6. doi: 10.1093/gigascience/giw012 (PMC5530312; doi:10.1093/gigascience/giw012)
Supplement: Reviewer_1_Report_(Original_Submission).pdf [file giw012_Reviewer_1_Report_(Original_Submission).pdf]

Reviewer Report

Title: "Whole genome sequencing of Chinese clearhead icefish, Protosalanx hyalocranius"

Version: Original Submission **Date:** 9/12/2016

Reviewer name: Christiaan Henkel

Reviewer Comments to Author:

This manuscript describes the genome assembly of the intriguing Chinese clearhead icefish. Overall, the sequencing and assembly meet the standards for a genome based on Illumina technology, as do the annotation and validation.

There are a few issues I would like to ask the authors to clarify:

1) Is it correct that a full third of the original sequencing data was discarded (252.1 Gbp -> 169.0 Gbp)? I could not find the exact meaning of SOAPfilter settings. (I think this tool does not include k-mer-based error correction or read trimming?)

2) The reason I ask, is because the genome size calculations (lines 97-101) are incorrect. Given N = 10.5 billion, k-depth = 20, it is easy to see how the 525 Mbp genome size was derived. However, the formula is not G = N/k-depth, and there should have been only 2 billion original reads, so this is clearly not the read number. Calculating N using the correct formula (line 98), I get 525 million = N * (125-17+1)/20, so N = 96 million, which is also nowhere near the (filtered) number of reads. Was a subset used? (Also note that the formula is only valid if all reads are of identical length, therefore trimmed reads should be omitted). In any case, a k-mer depth of only 20 must be incorrect (or based on a subset) in itself, as the genome coverage (table 1) is 315x.

3) Line 106: it should be reported that the 536 Mbp in scaffolds contain 121.7 Mbp in gaps. Whether the assembly then still qualifies as high quality is debatable, this depends fully on whether the genome size is really expected to be 525 Mbp (in which case the assembly misses 21% of the genome - not high quality), or whether the genome size is actually much smaller and the gaps between contigs are artificially large because of uncertainties in read library insert sizes.

Typos:

Line 104: 'fulfilled' -> better 'filled'

Line 124: ReBase -> RepBase. Also, please fix the author list of the corresponding entry [19] in the references.

Line 142: 'six … genomes, including…' then lists all six.

Level of Interest

Please indicate how interesting you found the manuscript: An article whose findings are important to those with closely related research interests

Quality of Written English

Please indicate the quality of language in the manuscript: Acceptable

Declaration of Competing Interests

Please complete a declaration of competing interests, considering the following questions:

- Have you in the past five years received reimbursements, fees, funding, or salary from an organisation that may in any way gain or lose financially from the publication of this manuscript, either now or in the future?
- Do you hold any stocks or shares in an organisation that may in any way gain or lose financially from the publication of this manuscript, either now or in the future?
- Do you hold or are you currently applying for any patents relating to the content of the manuscript?
- Have you received reimbursements, fees, funding, or salary from an organization that holds or has applied for patents relating to the content of the manuscript?
- Do you have any other financial competing interests?
- Do you have any non-financial competing interests in relation to this paper?

If you can answer no to all of the above, write 'I declare that I have no competing interests' below. If your reply is yes to any, please give details below.

I declare that I have no competing interests

I agree to the open peer review policy of the journal. I understand that my name will be included on my report to the authors and, if the manuscript is accepted for publication, my named report including any attachments I upload will be posted on the website along with the authors' responses. I agree for my report to be made available under an Open Access Creative Commons CC-BY license (http://creativecommons.org/licenses/by/4.0/). I understand that any comments which I do not wish to be included in my named report can be included as confidential comments to the editors, which will not be published.

I agree to the open peer review policy of the journal
