# Supplementary material for: Whole genome sequencing of Chinese clearhead icefish, Protosalanx hyalocranius
Source: Gigascience. 2017 Feb 24;6(4):1–6. doi: 10.1093/gigascience/giw012 (PMC5530312; doi:10.1093/gigascience/giw012)
Supplement: Reviewer_1_Report_(Revision_1).pdf [file giw012_Reviewer_1_Report_(Revision_1).pdf]

Reviewer Report

Title: "Whole genome sequencing of Chinese clearhead icefish, Protosalanx hyalocranius"

Version: Revision 1 **Date:** 10/19/2016

Reviewer name: Christiaan Henkel

Reviewer Comments to Author:

I thank the authors for responding to the minor issues I raised, and for their inclusion of additional evolutionary analyses. I am satisfied with their responses, however I still have a few minor suggestions:

- Regarding the genome size calculation, thank you for the clarification. As I read the formula, it is now clear that this calculation is based on a subset of the data. This is of course fine (and wise), but to avoid confusion I would suggest explicitly stating this in line 88 or 95.

- Regarding the assembly size, I think the total gap length could be included in table 1.

As for the evolutionary analyses, these are interesting and appropriate, however in both cases I suggest rephrasing the final lines summarizing the results, as these are now slightly ambiguous.

- Line 185/186: 'the close relationship between clearhead icefish and zebrafish & medaka'. Zebrafish and medaka are themselves not closely related fish species at all. Perhaps it is better to simply state that the data demonstrate the phylogenetic position of the clearhead icefish.

-Line 206: 'clearhead icefish also experienced the WGD, and it appeared more recently than medaka'. This could be read as a more recent WGD than in the case of medaka, when it is of course the same WGD (at exactly the same time).

Level of Interest

Please indicate how interesting you found the manuscript: An article of importance in its field

Quality of Written English

Please indicate the quality of language in the manuscript: Acceptable

Declaration of Competing Interests

Please complete a declaration of competing interests, considering the following questions:

- Have you in the past five years received reimbursements, fees, funding, or salary from an organisation that may in any way gain or lose financially from the publication of this manuscript, either now or in the future?
- Do you hold any stocks or shares in an organisation that may in any way gain or lose financially from the publication of this manuscript, either now or in the future?
- Do you hold or are you currently applying for any patents relating to the content of the manuscript?
- Have you received reimbursements, fees, funding, or salary from an organization that holds or has applied for patents relating to the content of the manuscript?
- Do you have any other financial competing interests?
- Do you have any non-financial competing interests in relation to this paper?

If you can answer no to all of the above, write 'I declare that I have no competing interests' below. If your reply is yes to any, please give details below.

I declare that I have no competing interests

I agree to the open peer review policy of the journal. I understand that my name will be included on my report to the authors and, if the manuscript is accepted for publication, my named report including any attachments I upload will be posted on the website along with the authors' responses. I agree for my report to be made available under an Open Access Creative Commons CC-BY license (http://creativecommons.org/licenses/by/4.0/). I understand that any comments which I do not wish to be included in my named report can be included as confidential comments to the editors, which will not be published.

I agree to the open peer review policy of the journal
