# Supplementary material for: Whole genome sequencing of Chinese clearhead icefish, Protosalanx hyalocranius
Source: Gigascience. 2017 Feb 24;6(4):1–6. doi: 10.1093/gigascience/giw012 (PMC5530312; doi:10.1093/gigascience/giw012)
Supplement: Reviewer_2_Report_(Original_Submission).pdf [file giw012_Reviewer_2_Report_(Original_Submission).pdf]

Reviewer Report

Title: "Whole genome sequencing of Chinese clearhead icefish, Protosalanx hyalocranius"

Version: Original Submission **Date:** 9/14/2016

Reviewer name: Frederic Brunet

Reviewer Comments to Author:

The paper presented here provides efficient combination of different programs used to characterise the genome of the Chinese clear head icefish.
I only can regret that the basic results provided here are way too succinct to get a full appreciation for the reviewers and the readers, lately. As an example, we are left with an final average value for the total number of transposable elements in this genome, but we are left with no idea about the proportions of each TE subdivisions. No evolutionary values are provided. Teleost fish are known to have a extra round of whole genome duplication, this result was not searched, nor discussed. Synteny hasn't been considered as well.
The authors put forward the methods they use and they provide minimalist details of the results.
Why hasn't the homology annotation done using the tilapia and platyfish?
Having in hand such a genome could have been the opportunity for more results, to enhance the interest of this publication. As an example, some phylogenetical analyses of key genes.

Minor typos:
l15 Missing blank space
l42 no plural at cavefish (fishes only used when an exact number is provided)
l124 missing "p" in RepBase
l128 missing "o" in MaxPeriod
l151 we emplyed GLEAN (no need of article before GLEAN)

The bibliography has a strong record of missing names (e.g. ref 19 and 39, or layout problems, like in ref. 20, 21, 23)

Level of Interest

Please indicate how interesting you found the manuscript: An article of limited interest

Quality of Written English

Please indicate the quality of language in the manuscript: Acceptable

Declaration of Competing Interests

Please complete a declaration of competing interests, considering the following questions:

- Have you in the past five years received reimbursements, fees, funding, or salary from an organisation that may in any way gain or lose financially from the publication of this manuscript, either now or in the future?
- Do you hold any stocks or shares in an organisation that may in any way gain or lose financially from the publication of this manuscript, either now or in the future?
- Do you hold or are you currently applying for any patents relating to the content of the manuscript?
- Have you received reimbursements, fees, funding, or salary from an organization that holds or has applied for patents relating to the content of the manuscript?
- Do you have any other financial competing interests?
- Do you have any non-financial competing interests in relation to this paper?

If you can answer no to all of the above, write 'I declare that I have no competing interests' below. If your reply is yes to any, please give details below.

I declare that I have no competing interests

I agree to the open peer review policy of the journal. I understand that my name will be included on my report to the authors and, if the manuscript is accepted for publication, my named report including any attachments I upload will be posted on the website along with the authors' responses. I agree for my report to be made available under an Open Access Creative Commons CC-BY license (http://creativecommons.org/licenses/by/4.0/). I understand that any comments which I do not wish to be included in my named report can be included as confidential comments to the editors, which will not be published.

I agree to the open peer review policy of the journal
