# Supplementary material for: Whole genome sequencing of Chinese clearhead icefish, Protosalanx hyalocranius
Source: Gigascience. 2017 Feb 24;6(4):1–6. doi: 10.1093/gigascience/giw012 (PMC5530312; doi:10.1093/gigascience/giw012)
Supplement: Reviewer_2_Report_(Revision_1).pdf [file giw012_Reviewer_2_Report_(Revision_1).pdf]

Reviewer Report

Title: "Whole genome sequencing of Chinese clearhead icefish, Protosalanx hyalocranius"

Version: Revision 1 **Date:** 5/24/2017

Reviewer name: Frederic Brunet

Reviewer Comments to Author:

I have not much to say about the quality of the work mastered here by the authors to generate the assembly of another fish genome, but among the Stomatii, a taxonomic location not yet been investigated.

Sentence line 185-186 is not well written. The phylogeny does not match the statement. Maybe the authors should rather name the taxonomic groups rather than naming two species that twisted their meaning.

Sentence line 204-206. This sentence shows that the authors have been mislead. There was only one event of whole genome duplication at the base of the Teleost genome. The different average rate of evolution in the different species observed by gene comparison has a different meaning that the one stated here. In this case, it is more suitable to rephrase this sentence. See similar comparisons made in the trout genome paper by Berthelot et al. 2014. Therefore, fig.2 is just a glimpse of what can be done, and this interpretation is too scarce and misleading.

Besides, recently published genomes have a finer tuned gene descriptions as better annotated sequences and more genes are described. Oreochromis niloticus, the Nile tilapia is a good example. On the contrary, the medaka genome lacks several genes, found in several other fish species, and sometimes key genes.

One suggestion would be to find out the number of duplicates originating from the teleost fish duplications compared to what have been described in the other species.

What TRF stands for in Table 2?

Line 184 typo in the word "sequence".

several "&" instead of "and".

Level of Interest

Please indicate how interesting you found the manuscript: An article whose findings are important to those with closely related research interests

Quality of Written English

Please indicate the quality of language in the manuscript: Acceptable

Declaration of Competing Interests

Please complete a declaration of competing interests, considering the following questions:

- Have you in the past five years received reimbursements, fees, funding, or salary from an organisation that may in any way gain or lose financially from the publication of this manuscript, either now or in the future?
- Do you hold any stocks or shares in an organisation that may in any way gain or lose financially from the publication of this manuscript, either now or in the future?
- Do you hold or are you currently applying for any patents relating to the content of the manuscript?
- Have you received reimbursements, fees, funding, or salary from an organization that holds or has applied for patents relating to the content of the manuscript?
- Do you have any other financial competing interests?
- Do you have any non-financial competing interests in relation to this paper?

If you can answer no to all of the above, write 'I declare that I have no competing interests' below. If your reply is yes to any, please give details below.

I declare that I have no competing interests.

I agree to the open peer review policy of the journal. I understand that my name will be included on my report to the authors and, if the manuscript is accepted for publication, my named report including any attachments I upload will be posted on the website along with the authors' responses. I agree for my report to be made available under an Open Access Creative Commons CC-BY license (http://creativecommons.org/licenses/by/4.0/). I understand that any comments which I do not wish to be included in my named report can be included as confidential comments to the editors, which will not be published.

I agree to the open peer review policy of the journal
